# MIDDLE-AGED AND OLDER LATINOS’ SATISFACTION OF BAILAMOS LATIN DANCE PROGRAM

**DOI:** 10.1093/geroni/igz038.2620

**Published:** 2019-11-08

**Authors:** Guilherme M Balbim, Susan Aguinaga, Isabela G Marques, Jacqueline Guzman, David X Marquez, Priscilla Vasquez

**Affiliations:** 1 University of Illinois at Chicago, Chicago, Illinois, United States; 2 University of Illinois at Urbana-Champaign, Urbana, Illinois, United States; 3 University of Victoria, Victoria, British Columbia, Canada; 4 University of Illinois at Urbana-Champaign, Urbana-Champaign, Illinois, United States; 5 University of California San Diego, California, San Diego, California, United States

## Abstract

Older Latinos engage in low levels of leisure-time physical activity (LTPA). Dance is a culturally appropriate activity which can be used to increase LTPA levels. We examined middle-aged and older Latinos’ satisfaction with the revised BAILAMOS Latin dance program. Healthy and low actives middle-aged and older Latinos (Mage = 64.89±7.08) were randomized to a 4-month dance program (n=167) or health education (n=166). The dance program consisted of four Latin dance styles (Merengue, Bachata, Cha Cha Cha, and Salsa). Classes were held twice a week for one hour. A total of 113 participants completed the program. Participants completed a program evaluation about the 4-months program regarding time, duration, settings, instructor, and overall satisfaction. Items were evaluated on a 1 (strongly disagree/very bad) to 4 (strongly agree/excellent) Likert agreement scale. A total of 73 participants evaluated the 4-month dance program. Participants evaluated the program adequacy agreeing or strongly agreeing as far: time, duration and setting (96-98%); instructor’s enthusiasm, quality of instructions, and eager to help (96-100%); dance program’s progression and enjoyment (93-96%); difficulty level (59%). Participants reported they intended to keep dancing by themselves (93%) and would recommend the program to friends and family (98%). Many participants (88%) reported feeling physically excellent or good as a result of the program, 95% found the program excellent or good, and 100% thought the program was worth their time. Overall, the BAILAMOS program evaluation demonstrated high participants’ acceptability and satisfaction. Those results can promote sustained LTPA and provide initial evidence to translation into community settings.

